# Cholera at Allahabad

**Published:** 1883-11

**Authors:** 


### Cholera at Allahabad.

A communication from India, bearing the date of August 5th, announces an outbreak of cholera in the Royal Artillery station at Allahabad. "During the last few days," observes our correspondent, "there have been twelve cases amongst the native servants of the regiment, and four deaths. Four Europeans have been attacked; one died, and three are under treatment, dangerously ill. No case has yet occurred in the 81st Foot, but two cases are reported to-day in the Native Infantry Regiment. The weather is much cooler than it has been for some months past; still the shade-temperature varies from 80° to 90° daily, and the percentage of humidity from 70 nearly to saturation."—*The British Medical Journal.*

